# Moderate Ethanol Pre-treatment Mitigates ICH-Induced Injury via ER Stress Modulation in Rats

**DOI:** 10.3389/fnmol.2021.682775

**Published:** 2021-06-25

**Authors:** Peter Bor-Chian Lin, Po-Kai Wang, Cheng-Yoong Pang, Wei-Fen Hu, Andy Po-Yi Tsai, Adrian L. Oblak, Hock-Kean Liew

**Affiliations:** ^1^Stark Neurosciences Research Institute, Indiana University School of Medicine, Indianapolis, IN, United States; ^2^Department of Anesthesiology, Hualien Tzu Chi Hospital, Buddhist Tzu Chi Medical Foundation, Hualien, Taiwan; ^3^School of Medicine, Tzu Chi University, Hualien, Taiwan; ^4^Department of Medical Research, Hualien Tzu Chi Hospital, Buddhist Tzu Chi Medical Foundation, Hualien, Taiwan; ^5^Institute of Medical Sciences, Tzu Chi University, Hualien, Taiwan; ^6^Department of Pharmacology and Toxicology, Tzu Chi University, Hualien, Taiwan; ^7^Neuro-Medical Scientific Center, Buddhist Tzu Chi Medical Foundation, Hualien, Taiwan

**Keywords:** alcohol consumption, oxidative stress, chaperone proteins, neuroinflmmation, neuroprotection

## Abstract

Intracerebral hemorrhage (ICH) is a life-threatening type of stroke that disrupts the normal neurological function of the brain. Clinical studies have reported a non-linear J-shaped association between alcohol consumption levels and the occurrence of cerebral stroke. Specifically, alcohol intoxication increases stroke incidence, while moderate alcohol pre-conditioning decreases stroke frequency and improves outcomes. Although alcohol pre-consumption is likely a crucial player in ICH, the underlying mechanism remains unclear. We performed 1-h alcohol pre-conditioning followed by ICH induction in Sprague-Dawley (SD) rats to investigate the role of alcohol pre-conditioning in ICH. Interestingly, behavioral test analysis found that ethanol intoxication (3 g/kg) aggravated ICH-induced neurological deficits, but moderate ethanol pre-conditioning (0.75 g/kg) ameliorated ICH-induced neurological deficits by reducing the oxidative stress and proinflammatory cytokines release. Moreover, we found that moderate ethanol pretreatment improved the striatal endoplasmic reticulum (ER) homeostasis by increasing the chaperone protein expression and reducing oxidative stress and apoptosis caused by ICH. Our findings show that the mechanism regulated by moderate ethanol pre-conditioning might be beneficial for ICH, indicating the importance of ER homeostasis, oxidative stress, and differential cytokines release in ICH.

## Introduction

Intracerebral hemorrhage (ICH) is an acute disease that causes severe disability and death. ICH accounts for ~10–20% of all strokes (Feigin et al., 2009; Sacco et al., 2009), and the incidence of ICH in Asians (18–24%) is two times higher than the rate in Caucasians (8–15%) (Kannel et al., 1970; Broderick et al., 2007; Hong et al., 2013; Toyoda, 2013), which leaves some public health burden and high social costs. ICH presents as bleeding in the brain parenchyma, and the hemorrhagic hot zone mainly occurs within the putamen, which is a part of the dorsal striatum (Qureshi et al., 2001, 2009; Xi et al., 2006). The primary injury of ICH is caused by the hematoma expansion over the first 24–48 h (Kazui et al., 1996), which leads to mechanical disruption of the brain parenchyma and shows a correlation with neurological deterioration (Kazui et al., 1996; Brott et al., 1997). And the secondary injury of ICH is caused by coagulation, hemolysis, and hemoglobin breakdown (Kazui et al., 1996). The ICH pathology leads to the production of reactive oxygen species (ROS), proinflammatory cytokine release, and disruption of the endoplasmic reticulum (ER) homeostasis (Huang et al., 2002; Kitaoka et al., 2002; Wu et al., 2002; Liew et al., 2019).

ER is the intracellular organelle that facilitates the homeostasis of newly synthesized proteins, and the misfolded or unfolded protein in the ER lumen led to the ER stress response. The accumulation of the unfolded proteins in the ER lumen delayed the protein translation. It activated the degradation of the misfolded proteins by targeting them to the ubiquitin-proteasome pathway, which is known as unfolded protein response (Ellgaard and Helenius, 2003). In a previous study, we indicated that ICH injury caused neuronal apoptosis via the disruption of ER homeostasis (Liew et al., 2019). In the acute phase of ICH injury, increased proteasome activity enhances the degradation of chaperone proteins and worsens the disease pathology.

Several risk factors increase ICH incidence, including hypertension, smoking, oral anticoagulant, antiplatelet usage, and the important one, alcohol intake (Taylor and Combs-Orme, 1985; Monforte et al., 1990; Huang et al., 2002). However, alcohol intake shows both beneficial and harmful effects in adults. Moderate drinking is associated with a lower incidence of cardiovascular diseases such as stroke, while a higher level of alcohol use is associated with liver and kidney diseases (Mukamal et al., 2005; Collins et al., 2009). Also, alcoholism leads to blood pressure elevation and inhibition of platelet aggregation, which could promote excessive bleeding (Pletsch et al., 2014). Binge drinking also increases the risk of ICH by impairing coagulation and directly affecting the integrity of cerebral vessels (Qureshi et al., 2001, 2009). There is much evidence disclosing how binge drinking aggravates brain injury (Maynard and Leasure, 2013; Duncan et al., 2016). The studies above suggested that chronic alcohol consumption affected the ICH incidence and the disease injury; however, the acute effect of alcohol exposure in ICH is unclear.

In clinical practice, patients of cerebral hemorrhage caused by a traumatic injury usually accompanied with a prior alcohol consumption. The physician in the emergency room needs to monitor the blood alcohol concentration (BAC) to decide the suitable treatment for the patients. Patients with cerebral hemorrhage were often treated with drugs to reduce their blood pressure. The combined effect of hypotensive agents and blood alcohol may cause severe hypotension and worsen the disease outcome. Therefore, it is important to study the acute effect of alcohol consumption in ICH-dependent injury.

Previously, we found that a high dose of ethanol pre-conditioning (3 g/kg body weight, i.p.) significantly aggravates hemorrhagic volume, brain edema, BBB disruption, microglial activation, elevated oxidative stress, and neuroinflammation (Liew et al., 2016). The summation of these consequences remarkably augments neurological impairment and results in a higher mortality rate in ethanol-pretreated ICH rats. Here, we report that acute alcohol intoxication aggravates the ICH-induced injury in rats. However, the effect of moderate alcohol pre-conditioning in ICH rats remains unclear. In this study, we determined the effect of moderate alcohol pre**-**conditioning on maintaining normal ER functions in ICH rats.

## Materials and Methods

### Experimental Design

All the experimental procedures for animal care and preparation were performed humanely in accordance with the National Institutes of Health Guide for the Care and Use of Laboratory Animals, and experimental protocol (IACUC 106-22) was approved by the Affidavit of Approval of Animal Use Protocol Board at Buddhist Tzu Chi General Hospital. All the rats were maintained in the Laboratory Animal Center at Tzu Chi University and were housed under 12-h light/dark cycles with food and water available *ad libitum*.

Male Sprague-Dawley (SD) rats weighing 300–350 g were adopted for this study. These animals were divided into six groups: Saline + Sham group, EtOH (0.75 g/kg, i.p.) + Sham group, EtOH (3 g/kg, i.p.) + Sham group, Saline + ICH group, EtOH (0.75 g/kg, i.p.) + ICH group, and EtOH (3 g/kg, i.p.) + ICH group. The timeline of experimental design and n numbers in each cohort were indicated in the Supplementary Figure 1.

### ICH Models

As previously described (Liew et al., 2019), the male SD rats were anesthetized with isoflurane (induction with 4% and maintenance with 1.5%) and randomly subjected to induce ICH. A 2-mm-diameter burr hole was created on the skull before injecting bacterial collagenase VII-S (0.23 U in 1.0 μL sterile saline) into the right striatum of the rat (0.0 mm rostral to bregma, 3.0 mm lateral to the midline, 5.0 mm ventral to the dural surface) (MacLellan et al., 2008; Liew et al., 2012a,b). The injection rate was 0.2 μL/min, and the needle was kept in place for another 5 min to prevent backflow. The burr hole was sealed with bone wax after the injection. During recovery from anesthesia, body temperature was maintained at 37°C with a heating pad. Normal saline was injected instead of the collagenase in the sham group (Liew et al., 2019).

### Functional Assay (mNSS)

Modified Neurological Severity Score (mNSS) assesses the neurological abnormalities (Chen et al., 2008). As shown in Table 1, the mNSS is a composite test of the motor, sensory, beam balance, reflexes, and abnormal movement and was performed on day 1 before ICH (pretest) and on days 1 and 3 after ICH, respectively. Neurological functions were graded on a scale of 0–18 (normal score, 0; maximal deficit score, 18). The higher the scores, the more severe the injury. All the evaluations were performed by an investigator blinded to the experimental treatment scheme (Chen et al., 2008).

**Table 1:** Modified neurological severity scores (mNSS) (Chen et al., 2008).

**Tests**	**Scores**
**Raising the rat by the tail**	
Flexion of forelimb	1
Flexion of hindlimb	1
Head moving more than 10° (vertical axis)	1
**Placing the rat on the floor**	
Inability to walk straight	1
Circling toward the paretic side	1
Falling to the paretic side	1
**Sensory tests**	
Visual and tactile placing	1
Proprioceptive test (deep sensory)	1
**Beam balance tests**	
Grasps side of the beam	1
Hugs the beam and one limb falls from the beam	2
Hugs the beam and two limbs fall from the beam or spins on beam	3
(>60 s)	
Attempts to balance on the beam but falls off (>40 s)	4
Attempts to balance on the beam but falls off (>20 s)	5
Falls off: no attempt to balance or hang on to the beam (<20 s)	6
**Reflexes (blunt or sharp stimulation) absence of:**	
Pinna reflex (head shakes when touching the auditory meatus)	1
Corneal reflex (eye blinks when lightly touching the cornea with cotton)	1
Startle reflex (motor response to a brief loud paper noise)	1
Seizures, myoclonus, myodystonia	1
**Maximum points**	18

### Lesion Volume

Morphometric measurement can be used as a good indication of the hemorrhagic volume on day 3 post-ICH (Liew et al., 2012b). Rat brain tissues were exposed and serially sliced (2 mm thickness) after decapitation. Brain slices were placed on the grid (0.5 ^*^ 0.5 cm^∧^2) and pictured. Images of the serial slices were then used to measure the hemorrhagic area in the striatum and quantitated the lesion volume (area ^*^ thickness) with Image J (NIH, Bethesda, MA).

### BAC, Liver Function, and Physiological Parameters

Rats were randomly assigned for the evaluation of BAC, the biochemical analysis of liver function [glutamate oxaloacetate transaminase (GOT)/glutamate pyruvate transaminase (GPT)], and physiological parameters, including mean arterial blood pressure (MABP) and blood gases.

The femoral vein was cannulated for the supplement of body fluid and drawn blood for BAC assay and liver function assay. BAC was measured on serum samples using an alcohol oxidase-based colorimetric assay kit (Cat. K620, BioVision Inc., Palo Alto, CA). BAC was measured before and 1, 2, and 3 h after treatment with EtOH. Contents of GOT and GPT) were examined as the liver function by using an automated dry chemistry analyzer (SP-4430; ARKRAY Inc., Kyoto, Japan).

The femoral artery was cannulated with a PE-50 polyethylene tube for monitoring MABP and blood gas. MABP was recorded through an amplifier (MP35; BIOPAC system, Goleta, CA) and stored in the computer. The MABP was measured before (baseline) and every 15 min until 4 h after treatment with EtOH. Blood gas was analyzed using the epoc® Blood Analysis System (Siemens Healthcare GmbH, Erlangen, Germany) and measured before and 4 h after EtOH treatment.

### Protein Carbonyl Assay (Oxidative Stress)

Protein carbonyl groups have been an important biomarker of oxidative stress (Dalle-Donne et al., 2003). The DNPH-tagged protein carbonyls are one of the most common methods to measure oxidative stress. Protein carbonyls were determined using a protein carbonyl content assay kit (Cat. ab126287; Abcam, Cambridge, the United Kingdom) according to the manufacturer's guidelines. As previously described (Liew et al., 2019), striatal samples were collected at 4 h after alcohol injection or 3 h after ICH injury. Striatal lysates containing 600 μg proteins were incubated with DNPH at room temperature for 10 min, and TCA solution was then being added to precipitate DNPH-tagged proteins. After centrifugation, pellets were washed with ice-cold acetone twice to remove free DNPH. Following the washes, guanidine solution was added to reconstitute DNPH. The O.D. absorbance at 375 nm was measured to determine the content of the DNPH by the spectrometer (OptiMax; Molecular Devices, San Jose, CA), which represents the content of protein carbonyls in striatal lysates.

### Total Ubiquitin Content (Unfolded Protein Response Marker)

Total ubiquitin (Ub) content was determined as an altered unfolded protein response. We used an enzyme-linked immunosorbent assay (ELISA) kit (Cat. abx576016; Abbexa Ltd., Cambridge, the United Kingdom) to reveal the amount of ubiquitin in protein lysates. As previously described (Liew et al., 2019), striatal lysates containing 100 μg proteins were incubated in wells pre-coated with the antibody specific to Ub at 37°C for 1 h. After washing away the unbound conjugates, a biotin-labeled antibody of Ub was incubated in wells for another hour at 37°C. The same washing procedures were performed again, and the avidin-conjugated horseradish peroxidase (HRP) was then added to each well and incubated at 37°C for 1 h. TMB substrate solution was added to produce a blue color product that changes into yellow after adding the acidic stop solution. O.D. absorbance at 450 nm was measured by the spectrometer (OptiMax; Molecular devices) to determine the concentration of ubiquitin.

### Proteasome Activity Assay

The activity of proteasome degradation in striatal lysates was measured according to the manufacturer's instructions (Proteasome Activity Fluorometric Assay Kit, cat. K245; Biovision). As previously described (Liew et al., 2019), after the extraction of tissue lysates from the striatum, protein concentration was then determined by Bio-Rad Protein Assay (Cat. 500-0006; Bio-Rad, Hercules CA). About 500 μg of total protein in striatal lysates was incubated in a 96-well plate at 37°C for 60 min with the succinyl-LLVY-AMC substrate. Striatal lysates were co-incubated with succinyl-LLVY-AMC and MG-132, which suppresses proteasome-related proteolytic activity and represents the non-proteasome activity. The amount of fluorescent AMC was measured with a spectrometer (OptiMax; Molecular devices) at 440 nm with an excitation wavelength at 380 nm.

### Reverse Transcription Quantitative Real-Time Polymerase Chain Reaction (RT-qPCR) Analysis

Total RNA was extracted from striatum samples by using an RNA isolation kit (Cat. NA021-100; GeneDireX, Inc., Vegas, NV). RNAs were then reverse-transcribed into complementary DNA through the SuperScript III First-Strand Synthesis kit (Cat. 18080-051; Invitrogen, Carlsbad, CA). One-step real-time RT-PCR analysis was performed to determine the expression of genes (Luminaris Color HiGreen qPCR Master Mix, K0391; Thermo Fisher Scientific, Waltham, MA) on the QuantStudio Real-Time PCR System (A34322; Thermo Fisher Scientific, Waltham, MA). The primer sequences are as follows: for spliced XBP-1 (NM_001271731.1), 5′-TGC TGA GTC CGC AGC AGG TG-3′ (forward) and 5′-GCT GGC AGG CTC TGG GGA AG-3′ (reverse); for total XBP-1 (both NM_001271731.1 and NM_001004210.2), 5′-AGC AGG TGC AGG CCC AGT T-3′ (forward) and 5′-TAC CAG ACT CTG GGG AAG GA-3′ (reverse), for Bip/GRP78 (NM_013083.2), 5′-GAC ATT TGC CCC AGA AGA AA-3′ (forward) and 5′-GCA ATA GTG CCA GCA TCC TT-3′ (reverse); for GRP94 (NM_001012197.2), 5′-CGA TGA AGT CGA TGT GGA TG-3′ (forward) and 5′-AGG CGA ACT TTT CCG ATT TT-3′ (reverse); for Hsc70/HSPA8 (NM_024351.2), 5′-GTT GCT TTC ACC GAC ACA GA-3′ (forward) and 5′-CAT CGT TCA CCA CCA TGA AG-3′ (reverse); for β-actin (NM_031144.3), 5′-TTG TAA CCA ACT GGG ACG ATA TGG-3′ (forward) and 5′-GAT CTT GAT CTT CAT GGT GCT AGG-3′ (reverse). The cycle threshold (Ct) value for each gene was normalized to the internal control gene (β-actin). The ratio of transcription on each gene was calculated as 2^**−ΔCt**^, where ΔCt is the difference of Ct (test gene) – Ct (β-actin).

### Western Blot Analysis

The ipsilateral striatum and the contralateral striatum were collected and rapidly frozen in liquid nitrogen and stored at −80°C until further analysis. Briefly, equal amounts of protein (40 μg) were loaded onto 10% sodium dodecyl sulfate–polyacrylamide gel electrophoresis (SDS–PAGE) gel, followed by blotting of the protein onto a polyvinylidene difluoride (PVDF) membrane (Immobilon-P PVDF membrane, Millipore, Bedford, MA), which was then blocked with 5% non-fat milk in 0.05% Tween–Tris-buffered saline. The membrane was probed with primary antibodies overnight at 4°C with gentle rotation. The primary antibodies and concentrations were used as follows: Bip/GRP78 (3177; Cell Signaling Technology, Inc., Beverly, MA; 1–1,000), GRP94 (Cat. ab13509, Abcam; 1–2,000), Hsc70 (Cat. ab51052, Abcam; 1–2,000), CCAAT-enhancer-binding protein homologous protein (CHOP) (2895; Cell Signaling Technology, Inc.; 1–2,000), and β-actin (A5441; Sigma-Aldrich, Saint Louis, MO; 1:10,000). Following washing and incubation with the respective HRP-conjugated antibodies (AP307P, and AP308P; EMD Millipore, Billerica, MA, 1:20,000) for 1 h at room temperature, and the HRP was then reacted with chemiluminescent ECL Plus Western blotting detection solution (RPN2133; Amersham Biosciences, Little Chalfont, the United Kingdom). The bands were visualized by exposure to X-ray films (34091; Kodak, Rochester, NY) and developed later. The intensities of bands were quantified with a densitometric analysis system (GS-800 Calibrated Densitometer, Bio-Rad, Hercules, CA) and calculated as the optical density × area of the band (Liew et al., 2015).

### Cytokine Assay

To examine the levels of proinflammatory cytokines in the striatal lysates, an equal amount of proteins (50 μg) were conducted to the ELISA of proinflammatory cytokines (IL-1β, IL-6, IL-10, and TNF-α) (Cat. DY501, DY506, DY522, and DY510, respectively) (R & D Systems, Minneapolis, MN, the United States according to the manufacturer's instructions (Liew et al., 2019).

### TUNEL Assay

The numbers of apoptotic cells were examined by using the *in situ* Cell death detection kit (Cat.11684817910, Roche, Basel, Switzerland) according to the manufacturer's instructions. After fixing and permeabilizing, brain slices were incubated with the terminal deoxynucleotidyl transferase (TdT) dUTP nick-end labeling (TUNEL) reaction mixture at 37°C for 1 h. Brain slices were rinsed with PBS and subsequently counterstained with DAPI. The apoptotic cells were visualized under a fluorescence microscope (Zeiss Axiovert 200 M; Carl Zeiss, Oberkochen, Germany) and manually quantified using ImageJ software (National Institutes of Health, Bethesda, MD).

### Statistical Analysis

Data were presented as mean ± S.E.M. All the statistical analyses were performed using SigmaStat software (Systat Software Inc., San Jose, CA) as repeated measures by one-way ANOVA followed by Tukey's test for *post-hoc* analysis. A *P*-value <0.05 was considered statistically significant.

## Results

### Moderate Ethanol Pre-conditioning Attenuates ICH-Induced Hematoma Lesion Volume and Neurological Deficit

**Figure 1:**
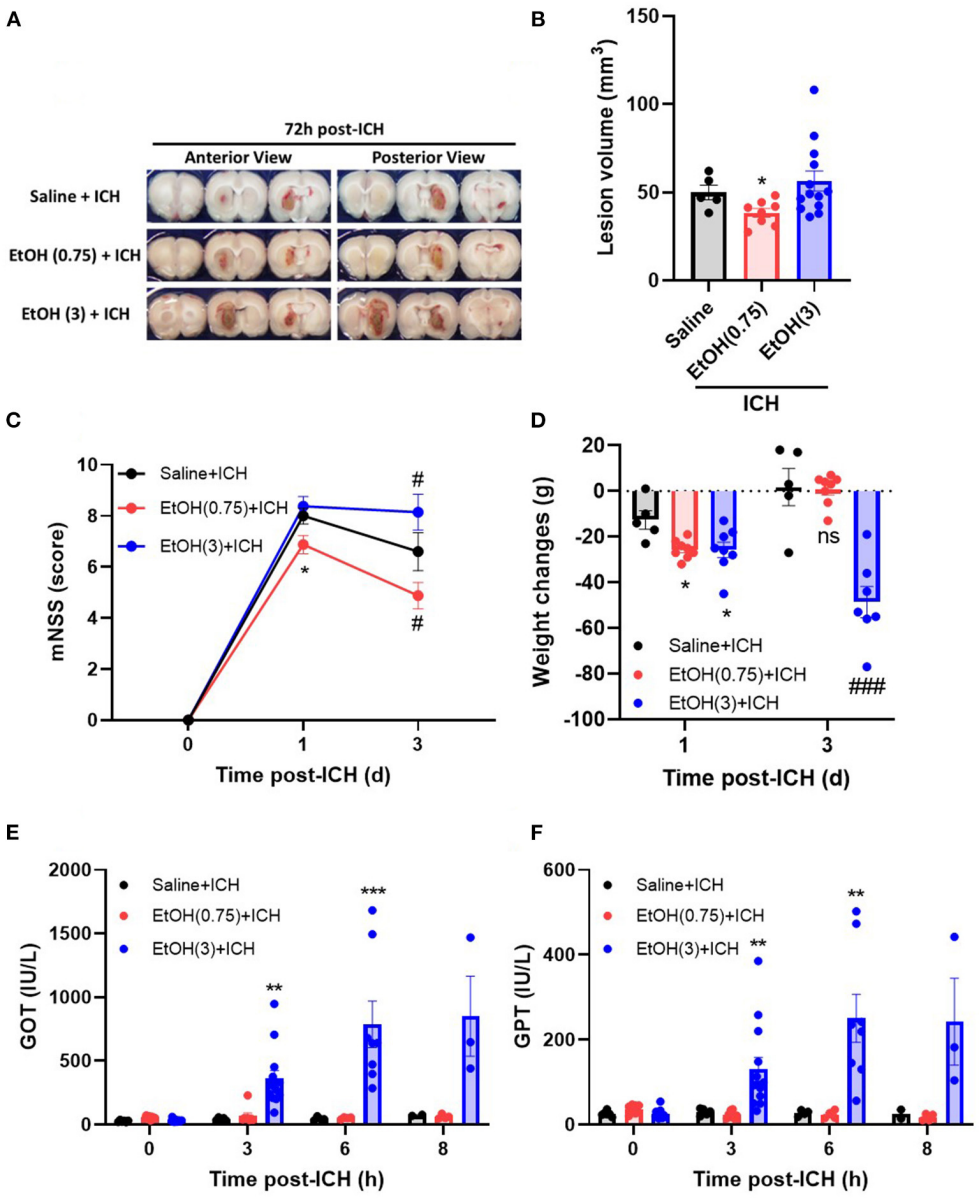
Effect of EtOH pre-conditioning on ICH-induced neurological deficits and liver functions. **(A,B)** Representative coronal sections **(A)** showed hemorrhagic brain areas, and lesion volumes **(B)** were determined by morphometric measurement on day 3 post-ICH. Values are shown as mean ± SEM (*n* = 8–14). *p < 0.05 in comparison with Saline + ICH group. **(C,D)** Modified neurological severity scores (mNSS) **(C)** and weight changes **(D)** were assessed on days 0, 1, and 3 post-ICH. Values are shown as mean ± SEM (*n* = 5–7). *p < 0.05 as compared to day 1 saline + ICH group. ^#^*p* < 0.05 as compared to day 3 saline + ICH group. **(E,F)** Serum levels of GOT **(E)** and GPT **(F)** were determined on 0, 3, 6, and 8 h post-ICH. Values are shown as mean ± SEM (*n* = 5–7). **p < 0.01 and ***p < 0.001 as compared to saline + ICH group. ^*###*^*p* < 0.001 as compared to saline + ICH group on day 3.

Moderate ethanol pre-conditioning (0.75 g/kg) attenuates hematoma volume on day 3 post-ICH (Figure 1A), but the high ethanol pre-conditioning (3.0 g/kg) exaggerated the hematomal lesion (Figure 1A). The lesion volume on day 3 post-ICH for EtOH (0.75) + ICH group was 38.4 ± 2.5 mm^**3**^, while Saline + ICH group and EtOH (3.0) + ICH group were 50.0 ± 4.2 and 60.0 ± 6.2 mm^3^, respectively (Figure 1B).

Moderate ethanol pre-conditioning also significantly reduced the ICH-induced neurological deficits (Figure 1C) and showed no effect on the body weight changes (Figure 1D) on day 1 post-ICH compared with the saline-treated group. In the Saline + ICH group, the mNSS scores were 8.0 ± 0.3 on day 1 post-ICH and decreased to 6.6 ± 0.7 on day 3. In the moderate ethanol group, the mNSS scores were reduced from 6.9 ± 0.4 (day 1) to 4.9 ± 0.4 (day 3) (Figure 1C). The high ethanol pre-conditioning group does not possess neurological recovery on day 3 post-ICH (Figure 1C).

As compared with the Saline + ICH group (−12.6 ± 4.0 g on day 1 and 1.8 ± 8.2 g on day 3), the body weight loss of both moderate and high ethanol pre-conditioning groups was elevated on day 1 (−25.6 ± 1.4 g), but the moderate ethanol group recovered on day 3 (0.75 ± 2.4 g) (Figure 1D). In contrast, the body weight loss of the high ethanol pre-conditioning group was significantly exaggerated on day 3 (−48.6 ± 6.4 g) post-ICH (Figure 1D).

An increased level of liver enzymes was observed in the ICH patients (Niizuma et al., 1988; Fujii et al., 1994; Meythaler et al., 1998); researchers concluded that the increased liver enzyme might cause the blood leakage readily and was attributed to the disease pathology. In this study, we found that both Saline + ICH and EtOH (0.75) + ICH groups did not have an affected liver function on either GOT (Figure 1E) and GPT (Figure 1F) activities during the acute phase of ICH (within 8 h post-ICH). However, the high ethanol pre-conditioning group showed significantly elevated the GOT (Figure 1E) and GPT (Figure 1F) level from 3 h and sustained to 8 h post-ICH.

### Effect of Ethanol Pre-conditioning on Physiological Parameters and BAC

**Figure 2:**
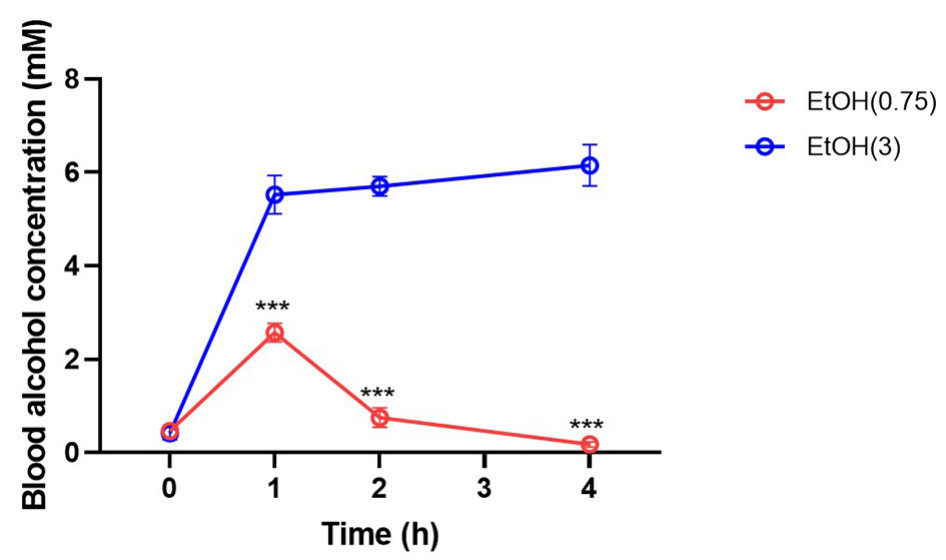
Effect of ethanol administration on blood alcohol concentration. Blood alcohol concentration was determined on 0, 1, 2, and 4 h post-administration by measuring the ethanol level in serum samples. Values are shown as mean ± SEM (*n* = 4). ***p < 0.001 as compared to EtOH (0.75) group.

Moderate ethanol pre-conditioning slightly reduced ~10 mmHg MABP on 1–2 h post-ethanol injection and recovered on 3 h (Supplementary Figure 2) after moderate ethanol pre-conditioning. The BAC was transiently elevated at 1 h post-moderate ethanol administration (Figure 2). However, high ethanol administration significantly reduced MABP and lasted for 3 h post-ICH (Supplementary Figure 2), accompanied by high BAC (Figure 2).

In the moderate ethanol group, oxygen saturation (SO_2_) was significantly elevated (Table 2). High ethanol pre-conditioning significantly augmented potassium (K^+^) and calcium (Ca^2+^) levels in serum (Table 2). In a previous study, we concluded that acute ethanol intoxication induced hemolysis in the brains of ICH rats (Liew et al., 2016). High ethanol pre-conditioning also elevated the amount of glucose and creatinine in serum.

**Table 2:** Physiological parameters in normal, EtOH (0.75 g/kg, i.p.), and EtOH (3 g/kg, i.p.) groups.

	**Normal**	**EtOH (0.75)**	**EtOH (3)**
**Blood Gases**			
pH	7.372 ± 0.043	7.444 ± 0.055^***^	7.376 ± 0.038
pCO_2_ (mmHg)	55.2 ± 7.2	42.4 ± 6.5^***^	45.1 ± 5.4^***^
pO2 (mmHg)	77.0 ± 13.5	92.6 ± 11.3^*^	75.8 ± 8.8
${\text{cHCO}}_{3}^{-}$ (mmol/L)	31.9 ± 2.1	28.8 ± 1.6^***^	26.3 ± 1.4^***^
BE(ecf) (mmol/L)	6.6 ± 2.0	4.7 ± 1.5^*^	1.1 ± 1.4^***^
ctCO_2_ (mmol/L)	33.6 ± 2.2	30.1 ± 1.7^***^	27.7 ± 1.6^***^
SO_2_ (%)	92.9 ± 4.1	97.1 ± 1.5^*^	94.0 ± 2.7
**Blood Electrolytes**			
Na^+^ (mmol/L)	140 ± 5	139 ± 3	137 ± 3
K^+^ (mmol/L)	3.8 ± 0.5	4.2 ± 0.3	4.5 ± 0.6^**^
Ca^2+^ (mmol/L)	1.30 ± 0.07	1.26 ± 0.06	1.11 ± 0.08^***^
Cl^−^ (mmol/L)	103 ± 6	104 ± 3	106 ± 1
AGap (mmol/L)	9 ± 3	10 ± 2	9 ± 2
**Hematology**			
Hct (%)	36 ± 2	37 ± 2	43 ± 4^***^
Hgb (g/dL)	12.3 ± 0.8	12.6 ± 0.7	14.8 ± 1.3^***^
**Blood Chemistry**			
Glucose (mmol/L)	8.8 ± 0.6	8.7 ± 0.4	10.4 ± 0.8^***^
Lactate (mmol/L)	0.70 ± 0.27	0.80 ± 0.14	0.85 ± 0.24
Creatinine (μmol/L)	47 ± 10	40 ± 9	68 ± 15^***^

*Arterial blood was drawn and analyzed before (normal group) and 4 h after ethanol administration (EtOH group). Values are shown as mean ± SD, calculated and analyzed by Student's t-test*.

* *p <0.05*,

** *p <0.01, and*

*** *p <0.001 as compared to normal group*.

We determined the BAC at 0, 1, 2, and 4 h post-ethanol pre-conditioning to evaluate alcohol metabolism. As a result, moderate ethanol pre-conditioning elevated BAC at 1 h post-injection and rapidly recover to the basal level at 2 h (Figure 2) post-ethanol administration. However, BAC remains high within 4 h in the high ethanol administration group.

### Moderate Ethanol Pre-conditioning Reduced ICH-Induced Oxidative Stress and ER Stress in the Striatum

**Figure 3:**
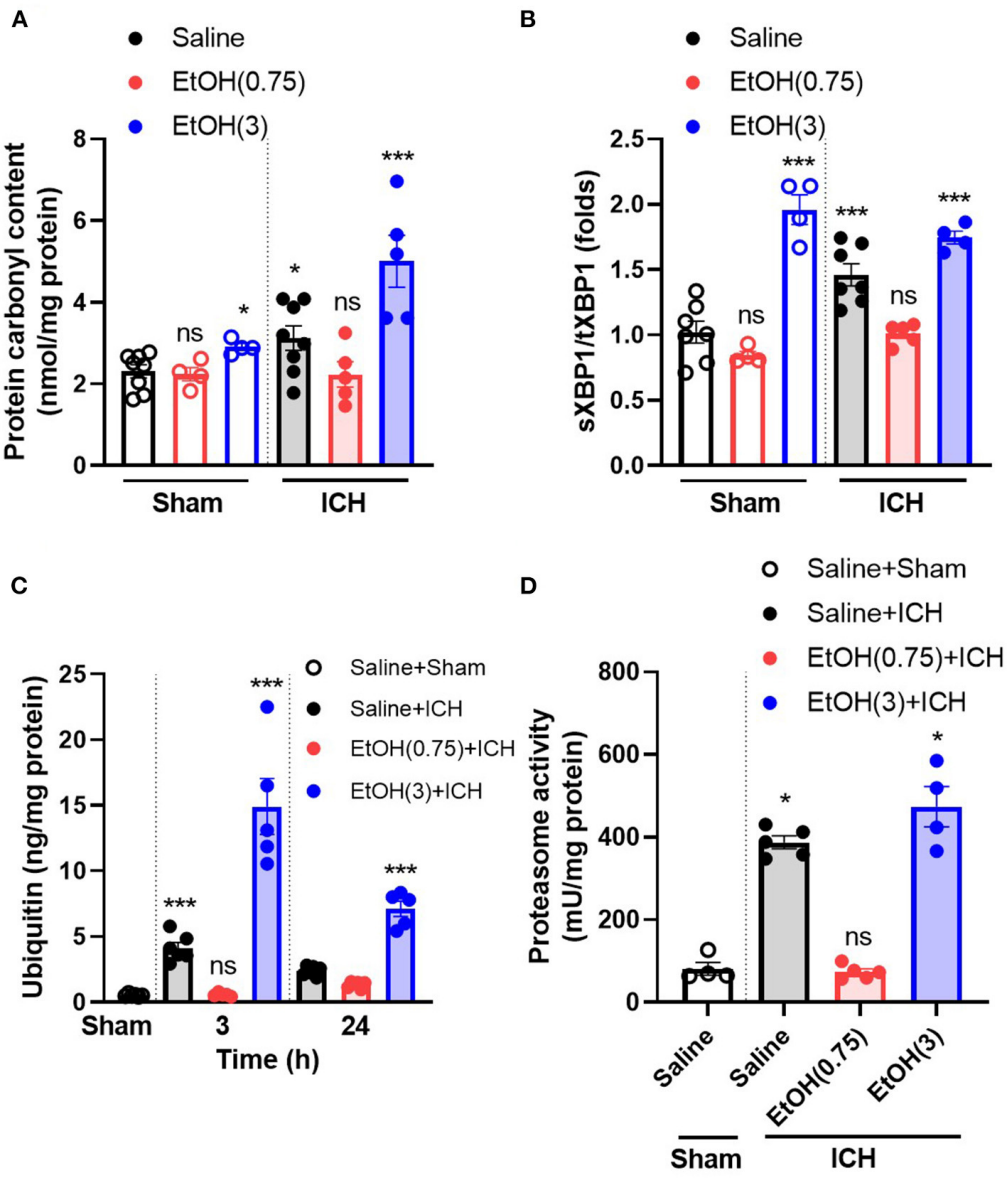
Moderate ethanol pre-conditioning restored the ICH-induced cytotoxicity and the disruption of protein homeostasis. **(A)** The level of protein carbonyl group was indicated as oxidative stress marker on day 1 post-ICH in each group (*n* = 4–8). *p < 0.05 and ***p < 0.001 as compared to sham group with ethanol administration. All the data were analyzed as repeated measures by one-way ANOVA followed by Tukey's test for *post-hoc* analysis. **(B–D)** The ratio of spliced XBP1 to total XBP1 (*n* = 4–7) **(B)**, ubiquitin content (*n* = 5) **(C)**, and proteasome activity (*n* = 4–5) **(D)** was indicated as the marker of unfolded protein response. ***p < 0.001 as compared to sham group.

ICH increased oxidative stress at 1 d (Figure 3A) in ipsilateral striatal tissue. Animal pre-treated with moderate ethanol significantly reduced the oxidative stress at 1 d post-ICH (Figure 3A). Also, as a marker of ER stress, the ratio of spliced XBP1 (sXBP1) to total XBP1 (tXBP1) induced by ICH significantly decreased by moderate ethanol pre-conditioning (Figure 3B). Moderate ethanol pre-conditioning prevented the ICH-induced elevation of ubiquitin content (Figure 3C) and proteasome overactivation (Figure 3D). All these factors above may be considered as a reduced unfolded protein response after moderate ethanol pre-conditioning. However, alcohol intoxication exaggerated oxidative stress and ER stress-related injury (Figure 3). Combined with these findings, the protective effect of moderate ethanol pre-conditioning on ICH may be due to reduced oxidative stress and relief ER stress.

### Moderate Ethanol Pre-conditioning Up-Regulated the Gene Expression of Chaperone Proteins

**Figure 4:**
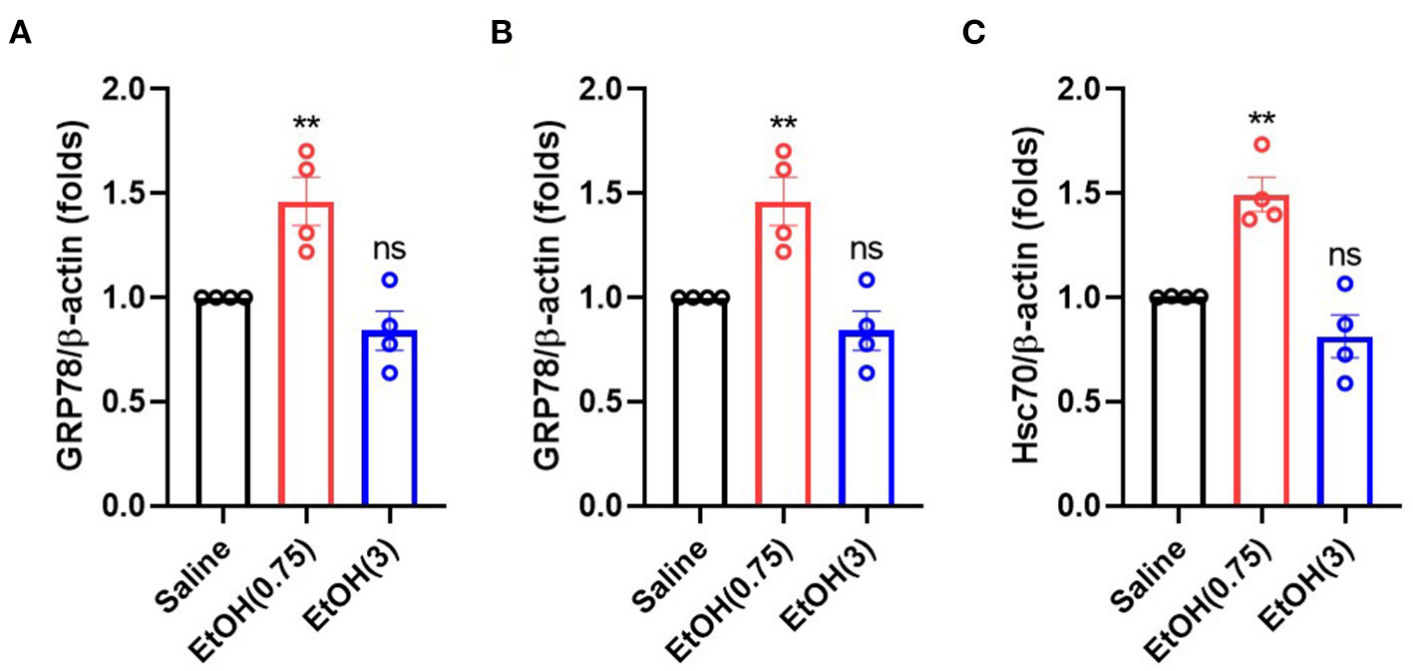
Ethanol administration regulated the gene expression of chaperone proteins. **(A–C)** mRNA level of GRP78 **(A)**, GRP94 **(B)**, and Hsc70 **(C)** on 3 h post-ethanol administration in each group. Values are shown as mean ± SEM (*n* = 4). **p < 0.01 as compared to animal with saline.

Alcohol treatment activates heat shock factor 1 (Hsf1) in cortical neurons, which binds to the alcohol response element (ARE, 5′-TCTGCGTCTCT-3′), and triggers the expression of several chaperone proteins, including GRP78, GRP94, and Hsc70 (Miles et al., 1991, 1994; Hsieh et al., 1996; Wilke et al., 2000; Pignataro et al., 2009). Here we examined GRP78, GRP94, and Hsc70's gene expression to determine the downstream signaling of moderate ethanol pre-conditioning on ER stress. As compared with the animal with saline treatment, moderate ethanol pre-conditioning significantly up-regulated the level of chaperone Bip/GRP78 (Figure 4A), GRP94 (Figure 4B), and Hsc70 (Figure 4C) mRNA in the striatum (1.29 ± 0.14-, 1.33 ± 0.01-, and 1.37 ± 0.01-fold, respectively). Meanwhile, EtOH (3) did not affect these gene expressions.

### Moderate Ethanol Pre-conditioning Eliminates ICH-Induced Disruption of Protein Homeostasis

**Figure 5:**
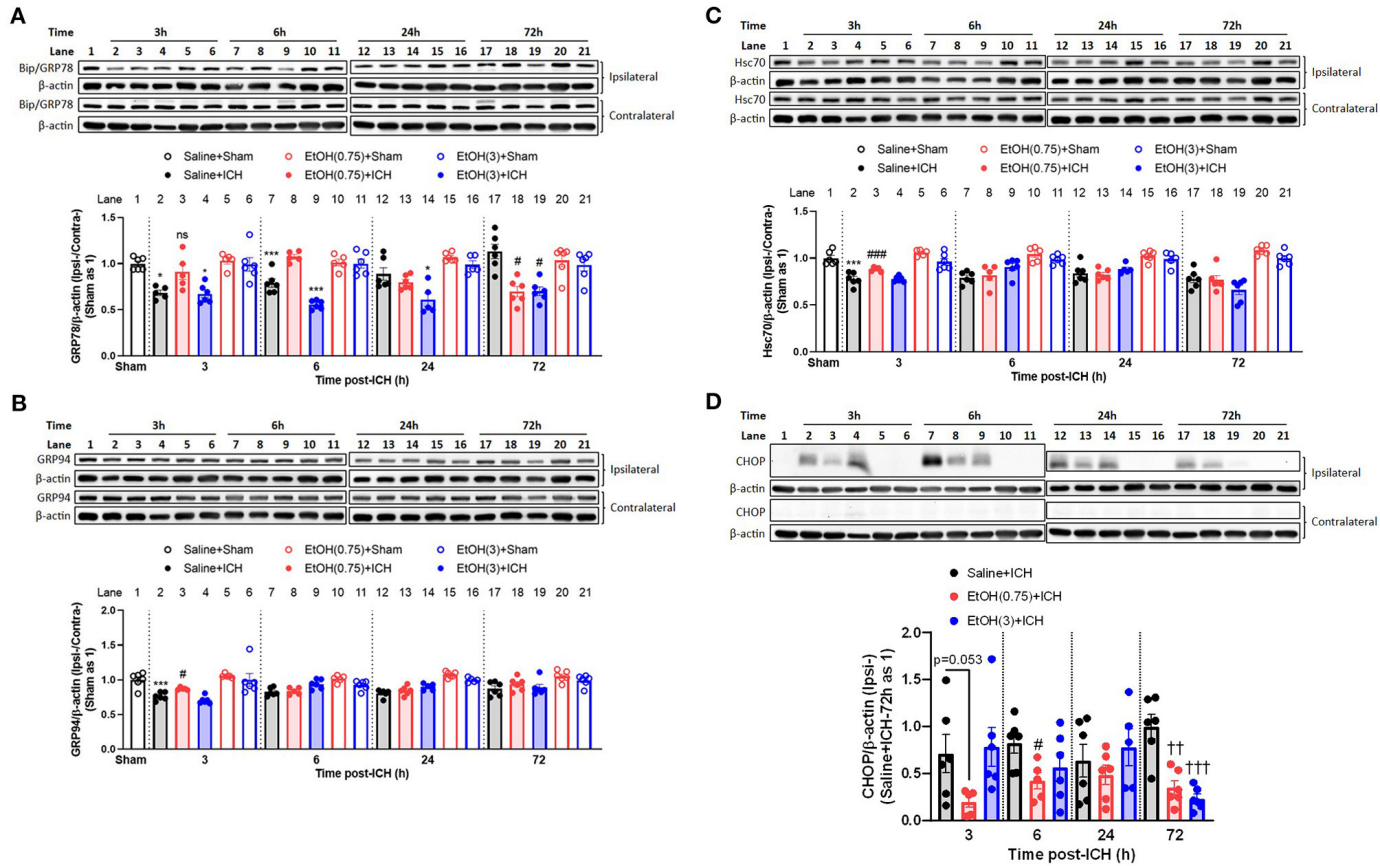
Moderate ethanol pre-conditioning restored the ICH-mediated reduction of chaperone protein and induction of CHOP protein. **(A–D)** Representative western blotting images showing bands of GRP78 **(A)**, GRP94 **(B)** and Hsc70 **(C)**, and CHOP **(D)** and quantitative analyses of each target protein detected in the ipsilateral and contralateral striatal lysates from each group and in each time point (3, 6, 24, and 72 h post-ICH). Values are shown as means ± SEM (*n* = 5–6). *p < 0.05, ***p < 0.001 as compared to normal group. ^#^*p* < 0.05 as compared to Saline + ICH group. ^††^*p* < 0.01 and ^†*††*^*p* < 0.001 as compared to saline + ICH group on 72 h post-ICH.

Our previous findings provided some evidence about the role of unfolded protein response on protein homeostasis in ICH (Liew et al., 2019). The proteasome degraded chaperone proteins, and this disruption of cellular homeostasis leads to apoptosis in neurons (Liew et al., 2019). Therefore, to study this mechanism on ethanol pre-conditioning, we quantified the content of chaperone proteins in the striatal lysates. Compared to the animal group with saline + sham injury (normal control, NC), ICH insults significantly increased the degradation of Bip/GRP78, GRP94, and Hsc70 (Figures 5A–C, black bar). Moderate ethanol pre-conditioning restored the degradation of Bip/GRP78, GRP94, and Hsc70 (Figures 5A–C, red bar). However, the high ethanol pre-conditioning-treated group did not restore the early degradation of chaperones Bip/GRP78, GRP94, and Hsc70 (Figures 5A–C, blue bar), respectively.

We also assessed the expression of CHOP, a downstream apoptotic protein of ER stress, in striatal lysates. In EtOH (0.75) + ICH group, ICH-induced CHOP expression was reduced (Figure 5D).

In the animal cohorts with sham injury, ethanol intoxication significantly increased the expression of chaperone protein in various timepoints (GRP78 in all timepoints, GRP94 in 3 h post-sham injury, and Hsc70 in 6 h post-sham injury; Supplementary Figures 3A–C). While the moderate ethanol pre-conditioning increased the expression of GRP78 in 72 h post-sham injury, and decreased the expression of Hsc70 in 3- and 24 h post-sham injury (Supplementary Figures 3A–C). There was no CHOP expression in all the animals with sham injury (Figure 5D).

### Moderate Ethanol Pre-conditioning Reduced Proinflammatory Cytokines in the Striatum

**Figure 6:**
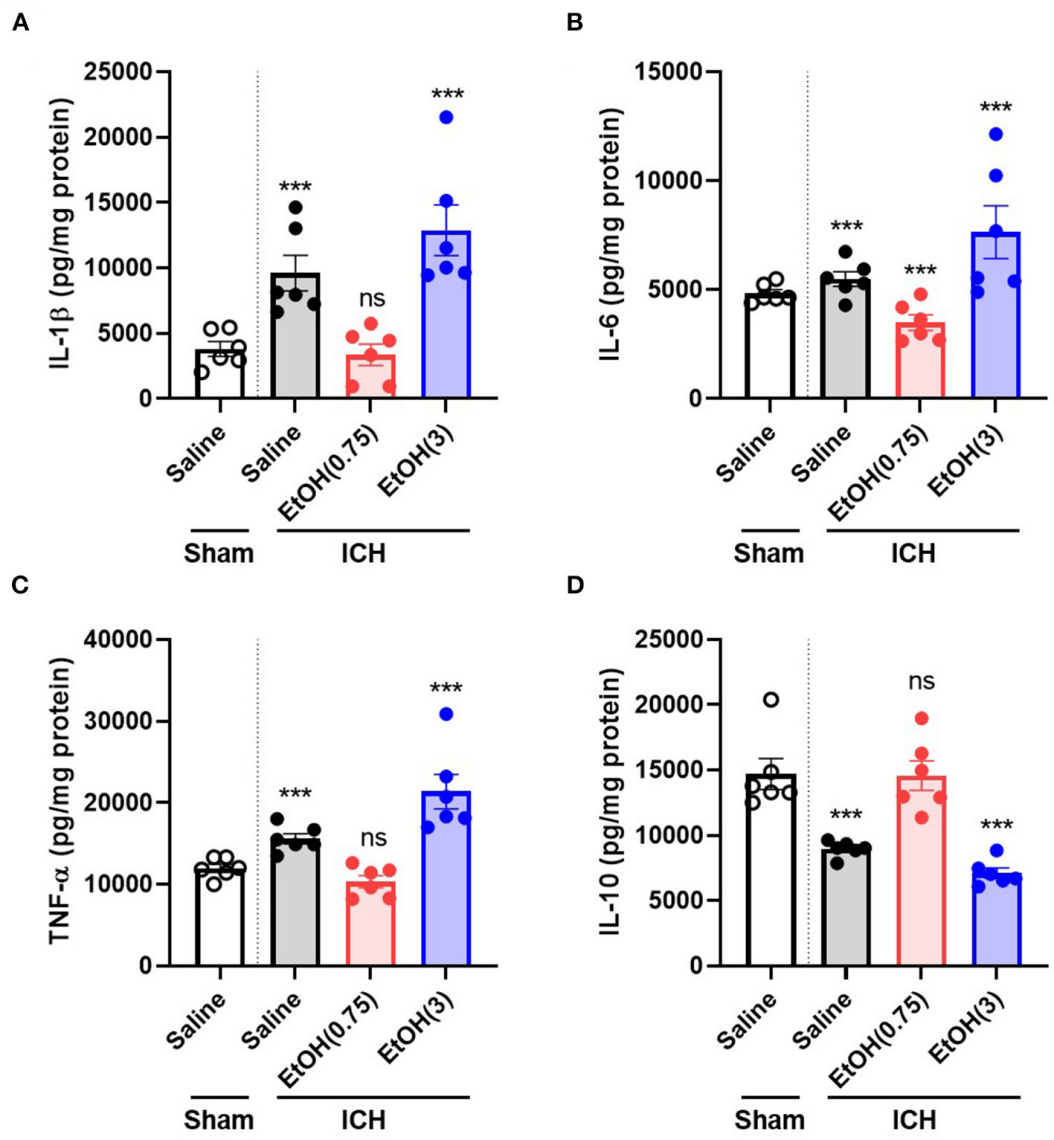
Moderate ethanol pre-conditioning altered proinflammatory cytokines release after ICH injury. **(A–D)** The levels of IL-1β **(A)**, IL-6 **(B)**, TNF-α **(C)**, and IL-10 **(D)** were measured on day 3 post-ICH in each group. Values are shown as means ± SEM (*n* = 6). ***p < 0.001 as compared to normal group.

Levels of proinflammatory cytokines were regulated in the striatum (up-regulated: IL-1β, IL-6, and TNF-α; down-regulated: IL-10) on day 3 post-ICH. In EtOH (0.75) + ICH group, elevated IL-1β, IL-6, and TNF-α levels were reduced (Figures 6A–C, respectively), and IL-10 level was restored (Figure 6D).

### Moderate Ethanol Pre-conditioning Reduced Apoptotic Cells in the Striatum

**Figure 7:**
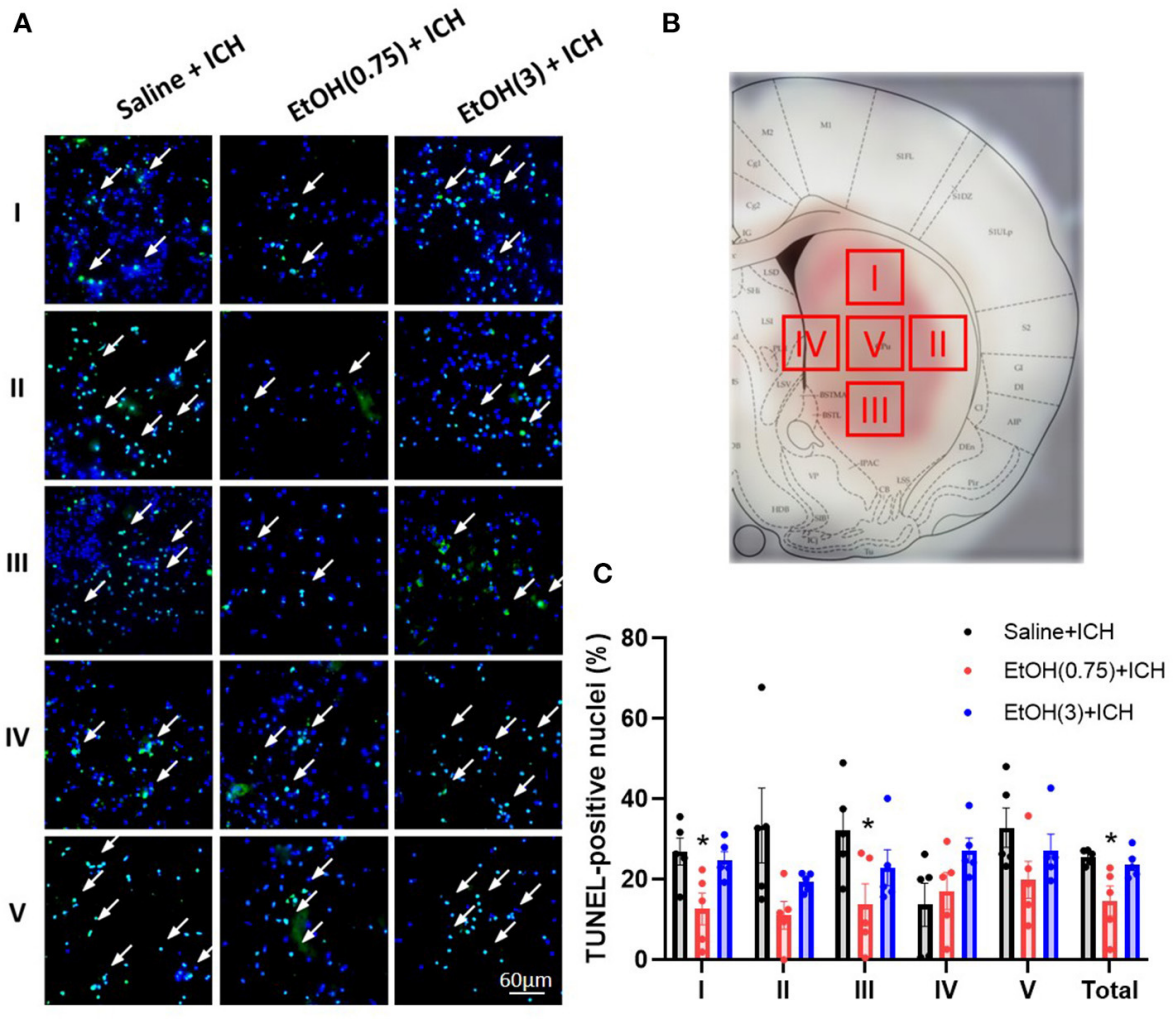
Moderate ethanol pre-conditioning reduced ICH-induced apoptosis in striatal regions. **(A,B)** Representative TUNEL-positive staining of apoptotic cells **(A)** in four regions around the peri-hematoma area (**B**, Regions I–IV) and core region (**B**, Region V). **(C)** The statistical measurement of ICH-induced apoptotic cells in each group. Values are shown as means ± SEM (*n* = 5). *p < 0.05 as compared to Saline + ICH group.

Finally, to quantify the level of apoptosis in the striatum, we examined the TUNEL assay in striatal sections. We measured the proportions of TUNEL-positive nuclei in the peri-hematoma region (Regions I, II, III, and IV) and core region (Region V) of hematoma in 1 d post-ICH (Figures 7A,B). Levels of TUNEL-positive nuclei in EtOH (0.75) + ICH group were significantly reduced in regions A, C, and total (Figure 7C). Meanwhile, there were no TUNEL-positive nuclei in the animal cohorts with sham injury (Supplementary Figure 4).

## Discussion

Epidemiologic studies suggest that light to moderate ethanol pre-conditioning reduces the risk of cardiovascular diseases (Mukamal et al., 2005; Collins et al., 2009). Moderate ethanol pre-conditioning can also ameliorate ischemia/reperfusion-induced brain damage by eliminating ROS production through NADPH oxidase (Wang et al., 2007). Moreover, the previous study suggested a reduced incidence of ICH on the patient with moderate drinking (odd ratio: 0.7; 95% *CI* = 0.4–1.2), indicating a crucial role of the moderate ethanol pre-conditioning in ICH (Thrift et al., 1999). Although alcohol pre-conditioning is likely a crucial player for ICH, the underlying mechanism remains unclear.

This study showed that moderate ethanol pre-conditioning attenuates ICH-induced injury by reducing oxidative stress and apoptosis while maintaining the ER-associated protein homeostasis. However, ethanol intoxication disrupted ER homeostasis and resulted in an aggravated ICH injury. Moderate ethanol pre-conditioning attenuates neurological deficit, and high ethanol pre-conditioning (intoxication) worsens the injury, suggesting the mechanism regulated by moderate ethanol pre-conditioning might be beneficial for ICH, and further highlights the importance of ER homeostasis, oxidative stress, and differential cytokines release in ICH.

In clinical practice, it is known that patient with alcohol consumption has an increased risk of ICH and enhanced neurological disorders (Casolla et al., 2012). A recent meta-analysis study showed that light and moderate drinkers (0–2 drinks/day) are associated with a reduced risk of ICH, in comparison with heavy drinkers (>2 drinks/day) (Larsson et al., 2016). Similar to this, we previously reported that ethanol intoxication delayed acute hematoma formation due to the hypotension caused by ethanol administration but accelerated BBB disruption via the activation of MMP-9 (Cheng et al., 2018). Here, we provide the evidence that moderate alcohol pre-conditioning decreased ICH-induced hematoma expansion (Figures 1A,B), behavioral deficits (Figure 1C, mNSS), and improved physiological outcome (Figure 1D, body weight changes). Meanwhile, the retained BAC in EtOH (3) group will elevate the liver enzyme level (Figures 1E,F) and oxidative stress (Figure 3A), which will enhance the ICH-induced injury in rats.

An increased level of liver enzymes in the ICH patients may attribute to the disease pathology by the prompt leakage of blood (Niizuma et al., 1988; Fujii et al., 1994; Meythaler et al., 1998). The elevated levels of the liver enzymes by high-alcohol pre-conditioning (Figures 1E,F) are related to reduced liver detoxification capacity, which will worsen the disease outcome by decreased ability against the oxidative stress in the brain. Also, as part of alcohol metabolism organs (Zimatkin and Deitrich, 1997), the increased acetaldehyde may aggravate the neurological deficits through the increased ROS in the brain.

We discovered that moderate alcohol pre-conditioning enhanced blood oxygen saturation and may result in a better prognosis by reducing oxidative stress in the brain parenchyma. In contrast, ethanol intoxication caused dehydration, results in the enhancement of hematocrit, hemoglobin, serum electrolyte, blood glucose, and blood creatinine level, which may worsen the ICH injury. Also, the elevation of the potassium level may be due to the hemolysis caused by acute ethanol intoxication (Table 2).

In our findings, the high alcohol administration caused hypotension, whereas the moderate pre-conditioning showed no effect on blood pressure (Supplementary Figure 2). Likewise, we reported that ethanol intoxication caused hypotension and led to delayed hematoma expansion (Cheng et al., 2018). The blood alcohol content was quickly metabolized after 2 h post-administration in the EtOH (0.75) group; however, the blood alcohol level remained high in the animal with higher ethanol administration (Figure 2). We suggested that the toxic effect driven by alcohol may not exist in the moderate alcohol pre-conditioning group when we induced ICH to the animal.

The GRP78 is a chaperone protein encoded by the HSPA5 gene, which locates in ER and helps to maintain the folding of newly synthesized proteins and the clearance of misfolded proteins by targeting these proteins to the unfolded protein response. The regulation of GRP78 has been reported as playing an important role in both ischemic and hemorrhagic stroke. The induction of GRP78 on ischemic pre-conditioned neurons leads to ER stress and autophagy (Zhang et al., 2015). Mild hypothermia-induced GRP78 reduction protects against ICH injury via attenuating ER stress and its downstream apoptosis in rats (Guo et al., 2016). Also, the regulation of GRP78 brings beneficial outcomes on both the *in vitro* and *in vivo* models of ischemic stroke (Ouyang et al., 2012). GRP94 is encoded by the HSP90B1 gene, which is also located in ER and plays a critical role in UPR. The *in vitro* model of ischemic stroke induces GRP94 expression, which regulates UPR and autophagy (Kim et al., 2003; Vavilis et al., 2016). Adenovirus-mediated antisense of GRP94 aggravates ischemic death of neuronal cells. However, adenovirus-mediated overexpression of GRP94 eliminates ischemic injury in SY5Y cells (Bando et al., 2003). Hsc70 is encoded by HSPA8 gene, a member of the heat shock protein 70 families. Hsc70 facilitates the proper folding of newly synthesized proteins and the degradation of mutant proteins. Hsc70 is located in the cytoplasm and lysosome and helps in the digestion of misfolded or unfolded proteins through the importation of these proteins into the lysosome in ICH (Niu et al., 2017). The content of Hsc70 is also elevated in the post-ischemic brain in either mRNA and protein levels (Kawagoe et al., 1992).

In our previous study, we concluded that ICH induced neurotoxicity by disrupting the brain protein homeostasis, causing augmented oxidative stress, unfolded protein response, and apoptosis (Liew et al., 2019). On the other hand, alcohol treatment has been identified with the ability to increase chaperone protein expression, including GRP78, GRP94, and Hsc70 (Miles et al., 1991, 1994; Hsieh et al., 1996; Wilke et al., 2000; Pignataro et al., 2009). Induction of these chaperone proteins mitigates neuronal injury (Kitao et al., 2001; Chen and Brown, 2007; Wang et al., 2010). Consistent with these studies, moderate ethanol pre-conditioning in our study mitigated the protein carbonyl content, which was increased during ICH injury (Figure 3A). The moderate ethanol pre-conditioning also reduced ER stress (Figure 3B) by maintaining brain homeostasis (Figures 3C–F).

We demonstrated that moderate ethanol pre-conditioning increases the expression of GRP78, GRP94, and Hsc70 in either mRNA (Figure 4) or protein (Figures 5A–C) level, which prevents the ICH-induced chaperone protein degradation, and further attenuates the ICH injury. Alternatively, ethanol intoxication increased chaperone protein degradation (Figures 5A–C), which augments neurological deficits. Also, moderate ethanol pre-conditioning reduced the apoptotic-related CHOP protein expression (Figure 5D), proinflammatory cytokine level (Figures 6A–D), and TUNEL-positive apoptotic cells (Figures 7A–C) in the striatum. All the findings above suggest overall beneficial effects on how the moderate alcohol pre-conditioning protects against ICH injury. We discovered an increased chaperone protein expression in the animals with ethanol intoxication (Supplementary Figures 3A–C); however, the enhanced expression was eliminated by the increased protein degradation in ICH injury.

On the other hand, we demonstrated that alcohol intoxication aggravated the ICH injury through an increased hematoma expansion (Figure 1B), elevated oxidative stress, and disruption of protein homeostasis (Figure 3), which leads to enhanced proinflammatory cytokine release (Figure 6) and results in worsening neurological deficits and behavioral outcomes (Figures 1C,D).

In clinical practice, physicians usually wait for sober before giving the ICH patient treatments because the interaction between alcohol and anesthetics may exaggerate the injury. However, in this study, we hint to clinics that depend on the blood ethanol level, indicating moderate alcohol level might benefit the hemorrhagic stroke.

**Figure 8:**
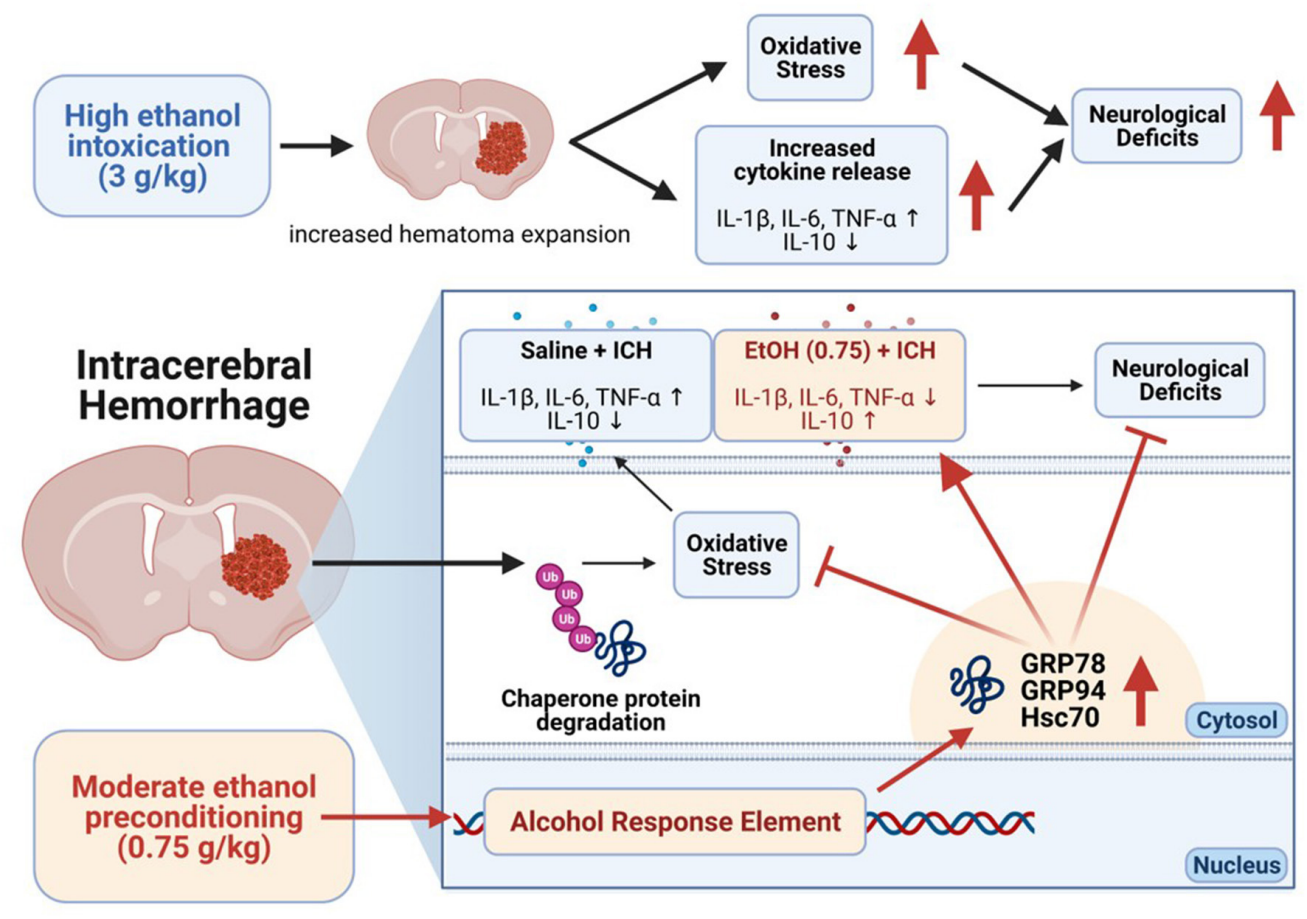
Overview mechanism of the moderate ethanol pre-conditioning in ICH-induced injury.

In conclusion, our results demonstrate that moderate ethanol pre-conditioning increased chaperone protein expression, promoting ER homeostasis, and further reduced oxidative stress and proinflammatory cytokines release. On the other hand, alcohol intoxication increased the hematoma expansion, oxidative stress, and cytokines release in ICH-induced injury (Figure 8). In this study, we focus on the acute effect of ethanol exposure to the ICH injury; however, animals with chronic alcohol exposure will have a better clinical relevance and needs further investigation. Also, future studies investigate the role of chaperone proteins in ICH that may allow for a novel therapeutic strategy of ICH.

## Data Availability Statement

The data that support the findings of this study are available from the corresponding author, Liew H-K, upon reasonable request.

## Ethics Statement

The animal study was reviewed and approved by Animal Use Protocol Board at Buddhist Tzu Chi General Hospital.

## Author Contributions

PL and H-KL involved in conceptualization. PL, W-FH, and H-KL involved in methodology. PL, W-FH, AT, and H-KL involved in validation. PL and W-FH involved in formal analysis. PL, P-KW, W-FH, and H-KL involved in investigation. P-KW, C-YP, and AO involved in providing resources. PL, P-KW, and H-KL involved in data curation. PL involved in writing the original draft preparation. PL, C-YP, AT, AO, and H-KL involved in writing the review and editing. PL and W-FH involved in visualization. AO and H-KL involved in supervision. H-KL involved in project administration. P-KW, C-YP, and H-KL involved in funding acquisition. All authors contributed to the article and approved the submitted version.

## Conflict of Interest

The authors declare that the research was conducted in the absence of any commercial or financial relationships that could be construed as a potential conflict of interest.

## Supplementary Material

Supplementary Figure 1 Overview of experimental design.

Supplementary Figure 2 Effect of ethanol administration on blood pressure. Blood pressure was measured every 15 min from 0 to 4 h of post-ethanol administration. Values are shown as mean ± SEM (*n* = 4). ^*^*p* <0.05, ^**^*p* < 0.01, and ^***^*p* < 0.001 as compared to EtOH (0.75) group.

Supplementary Figure 3 Ethanol pre-conditioning altered chaperone protein expression. **(A–C)** Protein expression of GRP78 **(A)**, GRP94 **(B)**, and Hsc70 **(C)** in the ipsilateral striatal lysates from each group and in each time point (3, 6, 24, and 72 h post-Sham injury). Values are shown as means ± SEM (*n* = 5–6). ^*^*p* < 0.05, ^***^*p* < 0.001 as compared to normal group.

Supplementary Figure 4 Ethanol pre-conditioning did not induce apoptosis in striatal regions. **(A–C)** Representative TUNEL staining in striatal area of sham animals. **(A)** Saline+Sham **(B)** EtOH(0.75)+Sham **(C)** EtOH(3)+Sham.
