# Supplementary figures and images for: Moderate Ethanol Pre-treatment Mitigates ICH-Induced Injury via ER Stress Modulation in Rats
Source: Front Mol Neurosci. 2021 Jun 25;14:682775. doi: 10.3389/fnmol.2021.682775 (PMC8267178; doi:10.3389/fnmol.2021.682775)

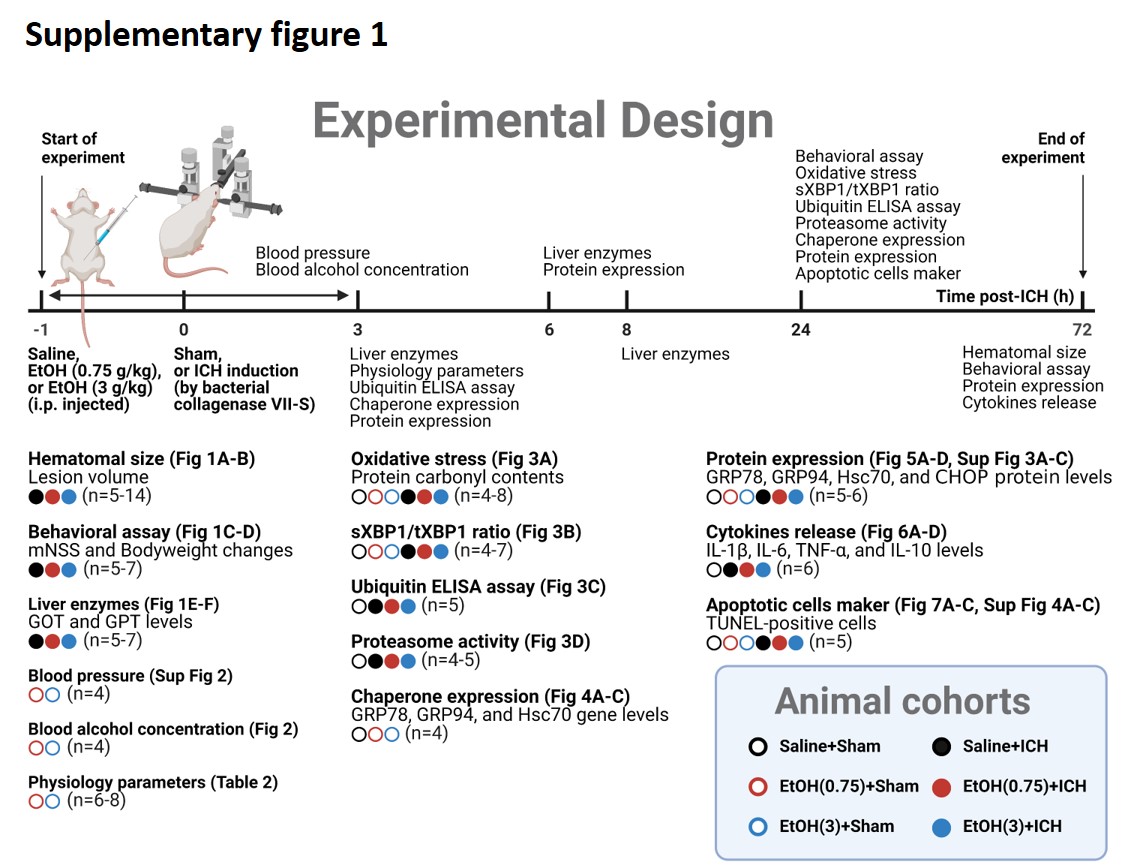

Supplement: Supplementary Figure 1 — Overview of experimental design. [file Image_1.jpg]

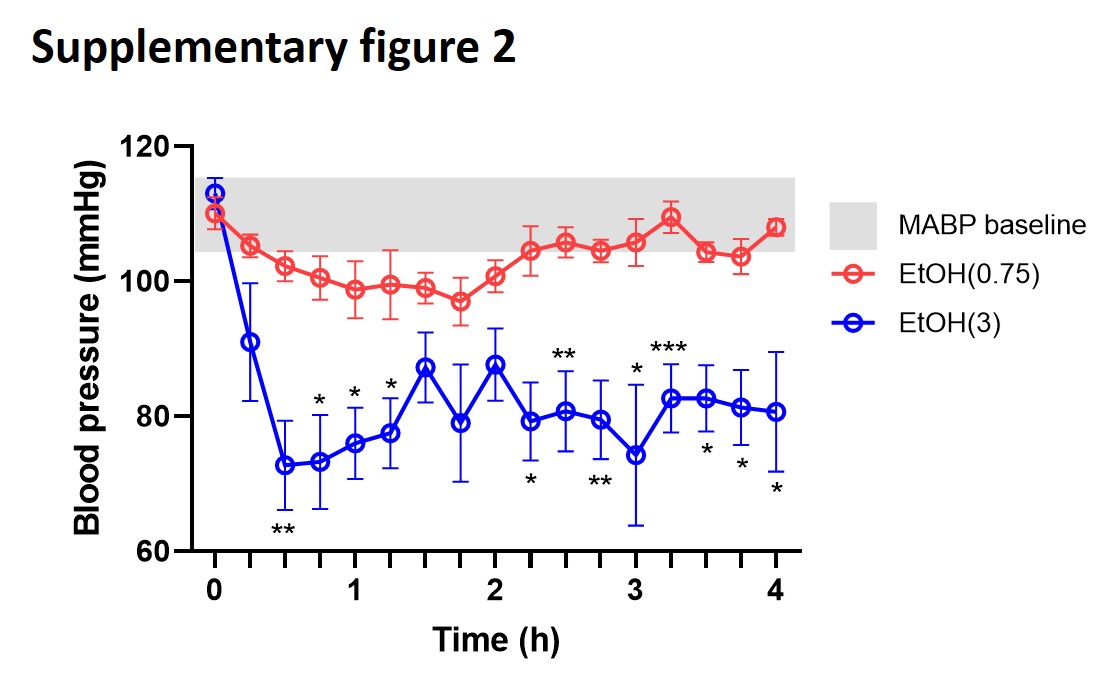

Supplement: Supplementary Figure 2 — Effect of ethanol administration on blood pressure. Blood pressure was measured every 15 min from 0 to 4 h of post-ethanol administration. Values are shown as mean ± SEM (n = 4). *p <0.05, **p < 0.01, and ***p < 0.001 as compared to EtOH (0.75) group. [file Image_2.JPEG]

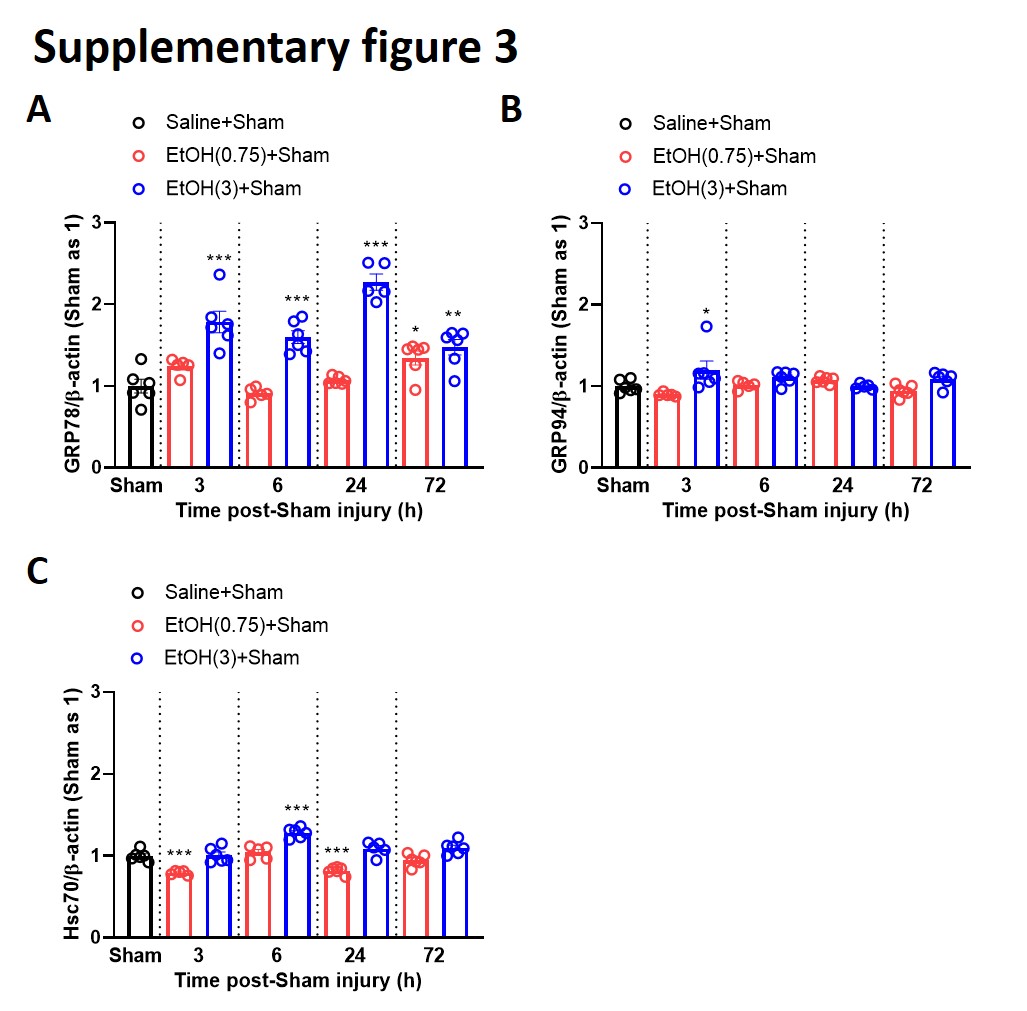

Supplement: Supplementary Figure 3 — Ethanol pre-conditioning altered chaperone protein expression. (A–C) Protein expression of GRP78 (A), GRP94 (B), and Hsc70 (C) in the ipsilateral striatal lysates from each group and in each time point (3, 6, 24, and 72 h post-Sham injury). Values are shown as means ± SEM (n = 5–6). *p < 0.05, ***p < 0.001 as compared to normal group. [file Image_3.JPEG]

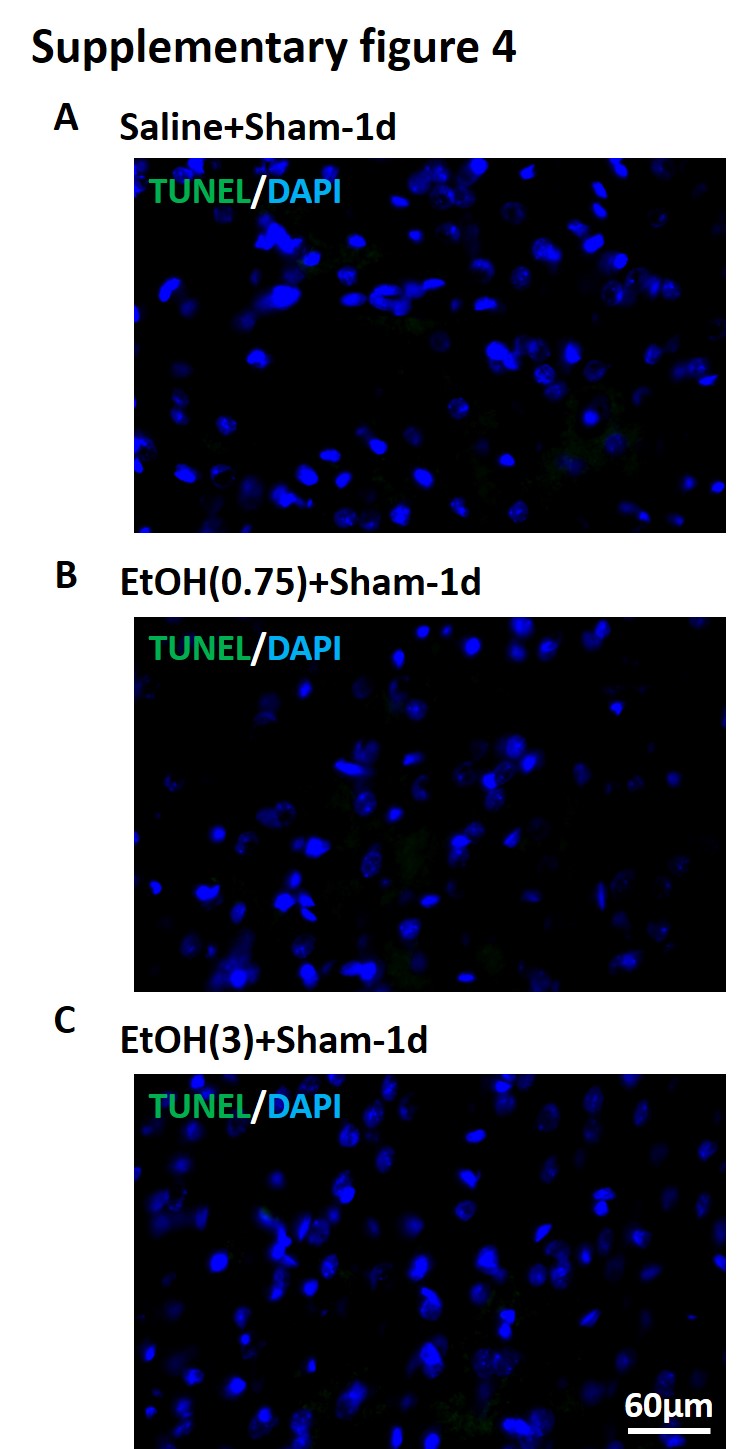

Supplement: Supplementary Figure 4 — Ethanol pre-conditioning did not induce apoptosis in striatal regions. (A–C) Representative TUNEL staining in striatal area of sham animals. (A) Saline+Sham (B) EtOH(0.75)+Sham (C) EtOH(3)+Sham. [file Image_4.JPEG]
